# Prevention of the sexual transmission of HIV-1: preparing for success

**DOI:** 10.1186/1758-2652-11-4

**Published:** 2008-10-01

**Authors:** Myron S Cohen, Pontiano Kaleebu, Thomas Coates

**Affiliations:** 1 Dept of Medicine, University of North Carolina, Chapel Hill, USA; 2 Medical Research Council/Uganda Virus Research Institute Research Unit on AIDS, Entebbe, Uganda; 3 The University of California, Los Angeles, USA

## Abstract

There are four opportunities for HIV prevention: before exposure, at the moment of exposure, immediately after exposure, and as secondary prevention focused on infected subjects. Until recently, most resources have been directed toward behavioral strategies aimed at preventing exposure entirely. Recognizing that these strategies are not enough to contain the epidemic, investigators are turning their attention to post-exposure prevention opportunities. There is increasing focus on the use of ART–either systemic or topical (microbicides)–to prevent infection at the moment of exposure. Likewise, there is growing evidence that ART treatment of infected people could serve as prevention as well. A number of ongoing clinical trials will shed some light on the potential of these approaches. Above all, prevention of HIV requires decision-makers to focus resources on strategies that are most effective. Finally, treatment of HIV and prevention of HIV must be considered and deployed *together*.

## Introduction

The 2007 UNAIDS report estimated that for every one person who receives antiretroviral treatment, 4–6 other people acquire HIV [1]. Yet, as has been recently noted [2], HIV prevention programs and initiatives have made only modest progress, and only in some communities. Furthermore, where there have been gains, they have not always been sustainable. HIV prevention can only succeed under the following conditions: i) all the available strategies are used in combination as "highly active prevention" [3]; ii) the menu of options are driven by scientific results and not ideology; iii) affected communities work together with organizations committed to prevention; iv) we continue use our growing knowledge of prevention–biological, structural and behavioral–to move past the social, economic, and other constraints we face today. Given the often limited prevention resources, we must focus on strategies that work [2]. In this article we review the documented successes and focus on recent data that is likely to shape near-term HIV prevention strategies.

## Prevention opportunities

**Figure 1:**
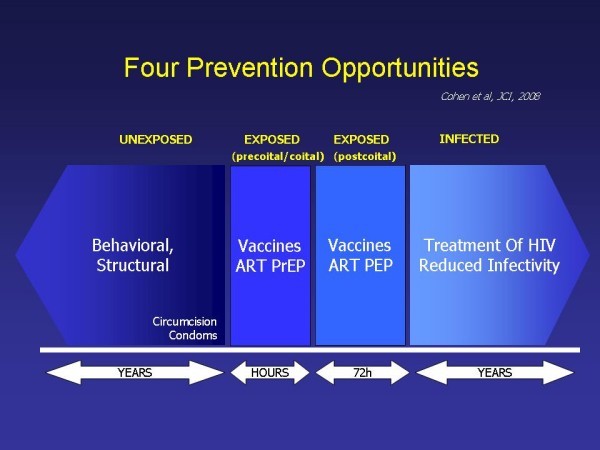
**HIV Prevention opportunities, adapted from**[**4**].

There are four separate and discrete opportunities for HIV prevention: before exposure to HIV, at the moment of exposure, immediately after exposure, and among people who are HIV infected [4] (Figure 1).

## HIV prevention before exposure

Behavioral interventions directed at those who are not infected with HIV must educate people about prevention, encourage access to services such as treatment for sexually transmitted infections or drug abuse, delay onset of first intercourse, increase condom use, and reduce the number of sexual partners and/or sharing of syringes and needles. Interventions can be deployed at the level of the individual, couple, family, peer group or network, institution, or community [3] (Table 1). Voluntary counseling and testing, a cornerstone of any HIV prevention strategy, can be applied at each of these levels: to individuals, to couples (as in Rwanda and Zambia), with entire families (as in Uganda), with peer groups (Thailand), and with entire communities (as in Project Accept).

**Table 1:** A multilevel approach to behavioural strategies for HIV prevention with HIV counselling and testing as an example.

Examples	Applied to HIV counselling and testing	
Individual	Education; drug-related or sexual risk reduction counselling; skills building; prevention case management	HIV testing and counselling for individuals35
Couple	Couples counselling	HIV counselling and testing for couples35–38
Family	Family-based counselling programmes	Home-based family HIV counselling and testing39
Peer group/network	Peer education; diffusion of innovation; network-based strategies	Voluntary counselling and testing for all network members
Institution (eg, school, workplace, prisons)	Institution-based programmes	Services for voluntary counselling and testing available within workplaces and other institutional settings40
Community	Mass media; social marketing; community mobilisation	Community-based voluntary counselling and testing (eg, Project Accept);41,42 Mobilisation and media to promote HIV counselling and testing

Adapted from [3].

Abstinence, be faithful, and condoms (ABC) has been the key message of the global HIV prevention effort, but political and religious influences have resulted in greater focus on abstinence only, despite the clear evidence that comprehensive approaches are far more effective [5]. As Collins and colleagues summarized,

It is time to scrap the ABCs and elevate the debate on HIV prevention beyond the incessant controversies over individual interventions. Small scale, isolated programs, however effective, will not bring the AIDS epidemic under control. To lower HIV incidence, especially in high transmission areas, policy makers, donors, and advocates need to demand national prevention efforts that re tailored to their epidemics, bring quality interventions to scale, and address environmental factors in vulnerability. That is why today's most commonly cited acronym for HIV prevention–"ABC"–falls severely short of what is needed to reduce HIV transmission. ABC infantilizes prevention, oversimplifying what should be an ongoing, strategic approach to reducing incidence [6].

## Barriers before exposure

Among the behavior change strategies for prevention of HIV is the use of mechanical barriers during sexual intercourse. The benefits of male condoms have been thoroughly documented [7], but the drawback is that these devices need to be used properly and nearly 100% of the time. According to a Cochrane review, when used properly, condom effectiveness is around 85% [8]. As a result of prevention education efforts, a recent survey shows that young woman in sub-Saharan Africa report increased condom by their male partners [9]. Likewise, female condoms have been shown to be an effective barrier against transmission of STIs–including HIV–but they have gained little popularity since their introduction [10].

In 2007, a study was conducted on the use of diaphragms to prevent HIV acquisition [11]. Because the endocervix is so rich in cells receptive to HIV [12], researchers had reason to believe that a diaphragm would prevent HIV infection; however, this trial failed to demonstrate protection[11]. The study results could be due to HIV infection outside the cervix, because adherence was poor, or because concomitant condom usage in both arms of the study limited the ability to detect a benefit from the diaphragm [11].

More recently, male circumcision has been studied as a possible means for preventing HIV transmission. Circumcision essentially erects a permanent barrier against HIV through removal of the foreskin. The mucosal foreskin glans of the penis is rich in cells receptive to HIV infection [12]. Powerful observational data suggested that circumcised men were much less likely to acquire HIV, implying the glans is the main site of HIV acquisition in men. Three randomized controlled trials demonstrated a minimum of 60% reduction in HIV acquisition [13-15]. Consequently, circumcision has been sought for high-risk subjects, but the logistical challenges of providing enough procedures to make an immediate impact are daunting. Many infants born in resource-constrained countries lack access to safe circumcisions, and there has been a distinct lack of political will or patience to institute safe neonatal circumcision worldwide.

## Other sexually transmitted diseases: a reappraisal

Classical STDs amplify the transmission of HIV by increasing the genital tract viral burden (infectiousness) and increasing susceptibility to HIV [16]. Overwhelming epidemiologic evidence links classical STDs and HIV [17], especially STDs that are more ubiquitous (e.g. HSV-2, trichomonas), produce lifelong infection (HSV-2), and/or produce ulcers (HSV-2, syphilis). Recognizing that STDs play a critical role in transmission, commitment to their treatment for the prevention of HIV is essential. Unfortunately, the results of clinical trials using treatment of STDs for prevention have been disappointing [18].

To some extent, this is not surprising. STD treatment can only prove effective if just the right person is treated for just the right STD with effective antibiotics for the right period of time. Most recently, daily suppressive treatment of HSV-2 in people with established HSV-2 infection failed to reduce HIV acquisition [19,20]. However, a substantial number of subjects developed genital ulcer, and it seems unlikely that acyclovir, the antibiotic administered, reduced the subclinical inflammation that is likely key to HIV transmission. A trial to determine whether suppression of HSV-2 in HIV-infected subjects can decrease sexual transmission of HIV-1 within a discordant couples is in progress [21].

Still, the disappointing results of these trials should not deliver the wrong message. First, the treatment of STDs is critically important on its own merits [22]. Second, people with STDs are much more likely to have unrecognized HIV [23], including incident infection [24]. Third, people with STDs who remain HIV negative have demonstrated HIV risk behaviors that demand emergent prevention efforts. HIV and classical STDs represent one, not two problems, and the merging of these (currently) separate disciplines is critical to reducing the incidence of both.

## HIV prevention at the time of exposure: biology beyond barriers

**Figure 2:**
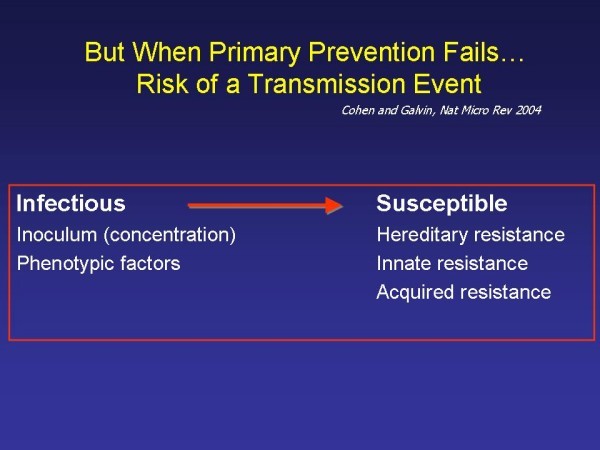
**Infectiousness and susceptibility, adapted from**[**16**].

If an HIV negative person has unprotected sexual exposure to an HIV positive person, transmission is possible. The transmission event is determined by the infectiousness of the "index case" and the susceptibility of "the host" [25](Figure 2). This topic has been extensively reviewed in the scientific literature [4]. The viral inoculum [26] and phenotype [27] play a critical role in transmission probability. The higher the concentration of the virus in the blood, the greater the probability of the transmission event [26,28]. In addition, the transmitted virus has unique properties: a single HIV variant launches sexually transmitted HIV infection 80% of the time [27], and the transmitted virus is generally capable of using the CCR5 receptor [27]. Conversely, polymorphisms and deletions in the CCR5 receptor reduce the probability of HIV acquisition [29]. In addition, the transmitted virus appears to be less well defended against antibody attack (i.e. a reduced glycan shield) [30].

Studies with HIV transmission in macaques show that both cell free (Miller C et al. JV 2005) and cell associated [31] HIV can be transmitted through vaginal exposure. In the presence of an ulcer, infection of mucosal cells and spread to lymph nodes is inordinately fast [31]. In humans, the probability of HIV transmission is likely amplified by sexually transmitted diseases that cause ulcer [16,32].

Beyond mechanical barriers, there are only two ways to prevent HIV infection at the moment of exposure: a credible host defense and/or antiviral therapy.

## Development of a protective HIV vaccine

The limits of vaccine development have been extensively discussed [25,33]. Three immune options have received the greatest attention: innate immunity, humoral immunity (antibodies) and/or cell-mediated immunity (cytotoxic lymphocytes) [34].

Antibodies have the capacity to block the attachment of HIV to receptive cells, or to neutralize HIV [35]. Indeed, a group of monoclonal antibodies which neutralize HIV-1 have been identified and described [36]. These antibodies have been used successfully in passive immunity experiments to protect infant rhesus macaques from peroral infection [37]. However, this type of neutralizing antibodies is not generally formed in vivo, perhaps because they are similar to autoantibodies that could harm the host [38]. HIV infection gradually leads to the formation of other antibodies [39] which form weeks or months after infection and after the creation of HIV mutants that uniformly escape the action of these antibodies [40]. Most of these antibodies will not neutralize heterologous viruses. However, a small number of hosts will develop broad neutralizing antibodies long after initial infection. The precise molecular mechanisms by which neutralizing broad antibodies limit HIV replication are not understood. Parenthetically, antibodies directed against HIV form too late in primary infection to explain reduction in viral burden [39].

**Figure 3:**
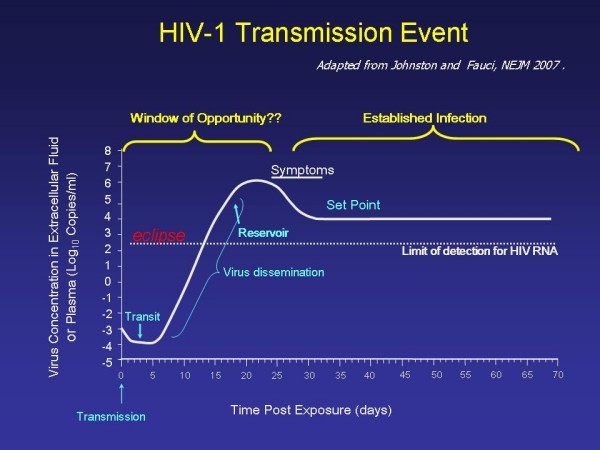
**HIV-1 Transmission Event, adapted from**[**25**].

The time between exposure to HIV, infection, and viral replication is very short (Figure 3) [39]. Even if a vaccine that evokes protective antibodies is developed, it will be a challenge for an anamnestic (memory) antibody response to evolve sufficiently enough to prevent transmission. Protection from HIV infection might require antibody generation at the mucosal surface (IgG or IgA) [41], and no mucosal vaccines have been developed. In addition, acute HIV infection compromises the B cell immune response required for antibody formation. About 30% of patients with acute HIV infection have Rhematoid factor detected, indicating disturbed function of B cells [39]. To date, one vaccine designed to stimulate antibodies has been tested, and no protection from infection was observed, although this vaccine did not generate systemic or mucosal neutralizing antibodies [42].

Given the difficulty in developing an antibody-based vaccine, investigators gravitated toward development of vaccines that stimulate cell-mediated immunity [34]. It has been demonstrated that i) animals with lymphocyte depletion cannot control viral replication; ii) some vaccines that stimulate a cell-mediated immune response limit the peak of viremia and transiently decrease the viral burden at "set-point" in macaques; and iii) improve animal survival [43]. While there is virtually no evidence that a T-cell based vaccine can prevent infection, it has long been argued that a reduced peak and set point HIV burden could prevent secondary transmission of HIV, and benefit the health and survival of the host.

A recent trial of a vaccine (Merck V520) that stimulated HIV responsive T cells failed to prevent HIV acquisition [44]. However, the vaccine also failed to reduce viral load at set point. Furthermore, more infections were observed in the group that received the vaccine than in the control group, but the reason for this phenomenon is not known [45]. Another ongoing trial that tests HIV proteins delivered in a canary pox virus vector and boosted with gp120 will be completed in 2009.

## ART for prevention

The use of antimicrobial agents to prevent the spread of infections has a long, broad and very successful history. It is surprising that to date, ART has not been more widely adapted as a prevention tool. ART is safe, available, becoming more affordable, and subject to structural modification(s) that might improve drug usage for public health purposes. While it is true that cost, toxicity, lack of adherence and viral drug resistance challenge the utility of the approach, it seems inevitable that ART will play a larger role in the HIV epidemic in the near future, but until recently this area of research has lacked a sense of urgency and adequate funding.

There are three ways ART could be employed: as pre-exposure prophylaxis (PreP), as post-exposure prophylaxis (PEP), and as prevention of secondary transmission from an infected person through suppression of viral concentration in the genital tract [46].

### Pre-exposure Prophylaxis (PreP)

Studies with animals have strongly suggested that antiretroviral drugs delivered topically (i.e. "microbicides") or systemically can prevent the transmission of HIV-1 [47-49]. A series of studies from the US Centers for Disease Control and Prevention used multiple rectal mucosal viral challenge on macaques who were given daily antiretroviral agents [50,51]. Tenofovir delayed SHIV_SF162P3 _infection, but after repeated exposure infection was prevented in only 1 of 4 animals studied [51]. However, high-dose tenofovir and emtricitabine given subcutaneously protected 6 of 6 macaques from infection [50].

Based on this animal data, trials of oral pre-exposure prophylaxis for uninfected high-risk individuals are now under way in Peru, Ecuador, Thailand, Botswana, and the United States. These trials use either tenofovir or a combination of tenofovir and emtricitabine [52] (Table 2). Results will emerge as early as 2010. It should be noted, however, that all of these trials offer prolonged (i.e. one year or more) daily dosing, interventions which are expensive and potentially toxic. It seems likely that prevention benefits will ultimately be realized with a briefer combination of pre- and post-exposure prophylaxis (see below), particularly since the most recent macaque studies suggest that optimally timed doses of PreP and a single dose of PEP are sufficient for protection.

**Table 2:** Current and Proposed Pre-Exposure Prophylaxis Trials, October 2007 Study (Sponsor) Study and Agent(s) (Dose) Population (Target N) Sites [52].

Study (sponsor)	Study and Agent(s) (Dose)	Population (Target N)	Sites
US CDC-NCHSTP-4323	Phase II daily TDF or daily oral placebo	MSM ages 18 to 60 (400)	US (anticipated completion 2009)
US CDC-NCHSTP-4370	Phase II/III daily TDF or daily oral placebo	IDU ages 20 to 60 (2,000)	Thailand (anticipated completion 2008)
CDC-NCHSTP-4940; BOTUSA MB06	Phase III daily Truvada or daily oral placebo	Men and women ages 18 to 29 (1,200)	Botswana (anticipated completion 2010)
iPrEX (NIAID/BMGF)	Phase III daily Truvada or daily oral placebo	MSM ages 18 and up (3,000)	Peru, Ecuador, Brazil, Thailand, South Africa, US (anticipated completion 2011)
FHI (USAID)	) Phase III daily Truvada or daily oral placebo	High-risk women ages 18 to 35 (3,900)	Kenya, Malawi, South Africa, Tanzania, Zimbabwe (study planned, no anticipated completion date yet)
Partners Study (BMGF)	Phase III daily TDF, daily Truvada, or daily oral Placebo	Discordant heterosexual couples ages 18 to 60 (4,000)	Uganda, Kenya (study planned, no anticipated completion date yet)
VOICE/MTN 003 (NIAID)	Phase IIB safety and effectiveness of daily tenofovir gel (1%) or placebo gel, or daily TDF (300 mg), Truvada, or oral placebo	Nonpregnant premenopausal women ages 18 to 35 (2,400 oral, 1,600 gel)	South Africa, Zambia, Malawi, Uganda, Zimbabwe (study planned, no anticipated completion date yet)

Antiretroviral therapy can also be delivered topically, via agents known as microbicides, a subject which has been extensively reviewed [53]. While the early days of microbicide research focused on drugs other than ART (e.g. detergents, surface active agents), a variety of more targeted biological products (antibodies and ART) are now being studied [53]. There are studies being conducted on antiviral agents including NNRTIs (s-DABO, TMC-120, U-781, and MIV-150), and the NRTI tenofovir is about to enter a phase 3 clinical efficacy trial.

Particularly exciting is the emergence of new, slow-release vaginal devices that might permit infrequent dosing of effective compounds [53]. One potential problem with topical ART is low-level, systemic absorption, which could promote antiviral resistance; however, in a completed PReP safety trial no women who acquired HIV developed mutations associated with tenofovir resistance [54].

### Postexposure prophylaxis (PEP)

The only prevention treatment option after unprotected HIV exposure is emergent use of antiretroviral agents [55]. Prophylaxis following occupational exposure to HIV is considered standard of care in the United States [56] and in most other countries [52]. This protocol was developed primarily from studies in macaques [57] and a single case control study of health care workers with needle stick exposures [58]. In the latter study, thirty-three healthcare workers who sero-converted following percutaneous exposure were compared with control subjects selected from six hundred and seventy-nine individuals who did not seroconvert after postexposure prophylaxis. Zidovudine (in a few cases, other antiretrovirals) given to individuals after percutaneous exposure to HIV led to an 81% risk reduction (CI, 48% to 94%) in HIV seroconversion.

Conducting randomized, controlled, clinical trials of postexposure prophylaxis to prevent the occupational or sexual transmission of HIV in humans are not feasible because of the inefficient transmission of HIV per sexual exposure, and the prohibitive cost of enrolling the very large number of subjects that would be needed to establish benefit. Current CDC guidelines recommend the use of 3 antiretroviral agents for 28 days following high-risk sexual exposure to a known or suspected HIV-infected partner [56]. Based on animal experiments it seems clear that prophylaxis should be administered urgently. In failures reported in a PEP registry delayed administration was a critical risk factor [59].

## Prevention for positives

Prevention for positives is only possible if a person knows his or her HIV status. Voluntary counseling and testing strategies (VCT), a cornerstone of HIV prevention, has generally been seen as a first defense against the spread of HIV disease, with the idea that a negative serological test, combined with prevention information, would inspire harm reduction [60-62]. Recognizing the critical role of knowledge of status, the US CDC and many other governments and organizations have recently moved to "opt-out testing" [63]. Others have championed the implementation of universal routinized testing [64].

One key and unresolved issue in preventing the sexual transmission of HIV is identifying the population most critical to the spread of the virus. Given the close relationship between viral load and transmission probability, one could assume that people with acute HIV and late infection (untreated) might be of greatest importance [65]. Indeed, in the only empiric study to address this issue a, substantial number of transmission events could be linked to index cases in precisely these stages of disease [26]. But a recent and compelling mathematical modeling exercise argued that subjects with unrecognized established infection with moderate viral loads in their blood (100,000 copies) are most critical [66], a finding which underscores the importance of knowledge of status.

A substantial number of couples are "discordant" (one partner is HIV-infected and the other is not). Recent massive household screening studies [21,67] have demonstrated that 49% of couples screened can be expected to be discordant, with some regional differences. Ongoing transmission within discordant couples occurs at a rate of about 8–11% per year, even in the face of counseling [68]. Thus the considerable danger of HIV transmission within untested discordant couples should not be underestimated [67,69].

These observations emphasize the importance of timely HIV detection and the need to develop effective counseling strategies to reduce the likelihood of secondary HIV transmission from people who know they are HIV infected. Studies on secondary transmission have raised the concern that ART treatment could actually increase the spread of HIV as a result of improved general health and increased libido after starting ART treatment [60]. Even more worrisome, this increase in secondary transmission would include resistant strains of the virus through patients who discontinue or fail therapy [60].

Recent studies have highlighted these concerns. Williamson et al. [70] found that HIV-infected men who have sex with men in the UK (and who knew their serostatus) had higher risk behaviors (including unprotected anal intercourse and intercourse) with partners of unknown or discordant serostatus than men who were negative or did not know their serostatus. Eisele et al. [71] found ongoing risk behaviors among men and women awaiting ART in Cape Town, South Africa; correlates of risk included failure to disclose serostatus and misconceptions about the relationship between ART and HIV transmission.

## ART for suppression of HIV

When used properly, ART can be expected to suppress HIV in the blood and the male genital tract [72]; suppression of HIV in the female genital tract appears to be less rapid and reliable [73]. However, STDs can increase shedding of HIV even in men [74] and women [75] receiving ART. While episodic viral shedding is easily documented, the risk of a transmission event during shedding is unknown.

There are three lines of evidence to suggest that ART reduces infectiousness of treated patients: retrospective analysis, prospective observational studies and ecological data. In two retrospective studies, HIV transmission was greatly reduced when the index cases in couples were offered therapy [76,77]. Two prospective observational studies had similar findings. In one study, of 1034 discordant couples in Zambia and Rwanda, the index partners in 248 couples were receiving ART [78]. Among the 42 partners in this cohort who acquired HIV since 2003, only 2 had partners receiving ART. A similar prospective observational study of Ugandan patients initiating ART reported a 98% reduction in the estimated risk of HIV transmission following the start of ART [79].

Retrospective and observational studies are susceptible to the effects of unexpected modifiers, including unmeasured sexual behavior(s) and condom use. In addition, the periods of observation generally cannot determine long term benefit or detect transmission of resistance viruses (see below). Perhaps most importantly, the studies only include index subjects who require ART for low CD4 counts or advanced HIV disease, whereas ART for prevention might wisely be employed at a much higher CD4 count, especially in people at greatest risk for transmitting HIV.

Several ecologic studies of the preventative benefit of ART have been completed. In a large closed cohort of homosexual men in San Francisco, California, a 60% reduction in anticipated cases of HIV was attributed to availability of ART for infected sexual partners [80]. A study from Taiwan showed a 53% reduction in the expected cases of HIV following the free provision of ART in 1997 [81]. More recently, a study in British Columbia, Canada suggested that up to 50% of expected incident HIV cases were averted by ART [82].

However, ecological prevention benefits of ART have not been universal. No reduction in incident HIV infections among men who have sex with men in San Francisco was observed despite widespread availability of ART [83], and increases in HIV incidence were found among homosexual men attending sexually transmitted disease clinics in Amsterdam, the Netherlands from 1991 to 2001 [84] regardless of treatment roll-out. Ecologic studies are greatly limited by an "ecologic fallacy": the inability to relate the patients who receive therapy to the actual incidence or prevalence of HIV in the community. In addition, the accuracy of HIV prevalence and incidence data in these many of these settings is unknown.

A randomized, controlled trial is underway to try to define the impact of ART on HIV transmission. HPTN 052 is designed to compare the effectiveness of two different treatment strategies to prevent the sexual transmission of HIV among 1750 serodiscordant couples [85]. HIV-infected partners with a CD4 count between 350–550 cells/mm^3 ^are randomly assigned to initiate ART at enrollment or to delay ART until their CD4 T-cell count falls below 250 cells/mm^3 ^or they develop an AIDS-defining illness. The results should detect a 35% reduction in HIV transmission to sexual partners due to ART treatment of HIV-infected subjects. In addition, this study compares the benefits of early versus delayed ART (ACTG 5245), a critical question if ART is going to be used more broadly as a public health tool.

## Rational selection of antiviral agents for prophylaxis or prevention

The number and choice of antiviral agents (whether systemic or topical) is vital to the success of any of the interventions discussed. The choice of ART regimen must also take into account the risk of HIV-resistant variants and the pharmacology of antiviral agents. The prevalence of de novo resistance in individuals with incident HIV infection differs greatly by country and region [86] but should be taken into account in selecting ART prophylactic regimens. Furthermore, resistance in the genital tract can be unique and sustained [87].

**Figure 4:**
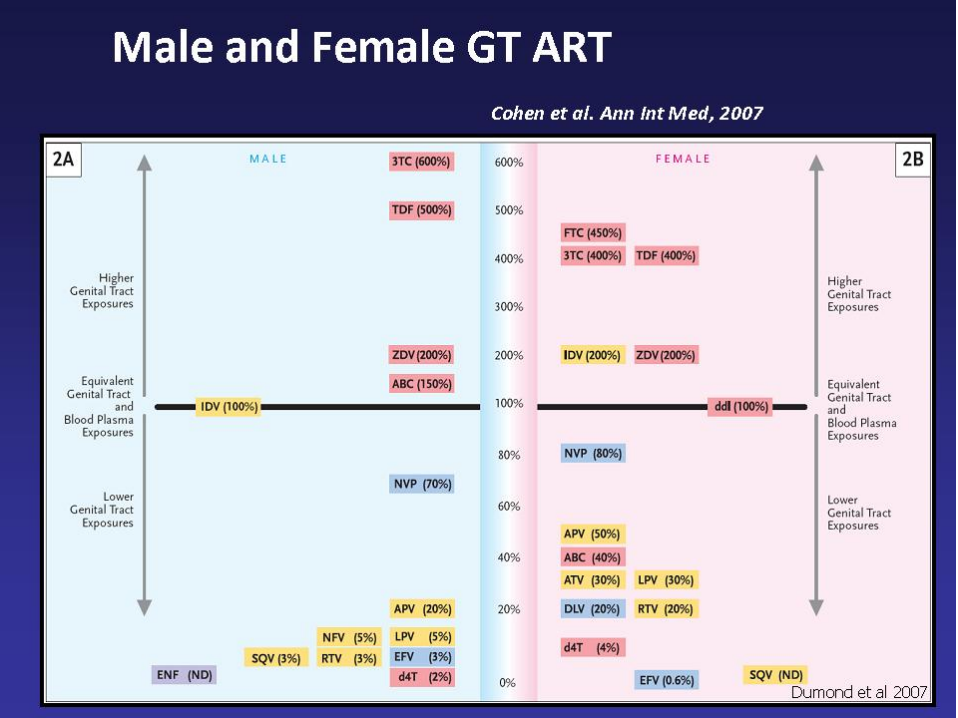
**Male and Female GT ART, reproduced with permission from**[**46**].

Recent findings on the pharmacology of antiretrovirals in the genital tract suggest that certain antiretroviral agents may be preferable for the prevention of HIV following sexual exposure (Figure 4). Lamivudine, emtricitabine, zidovudine, tenofovir and maraviroc concentrations in the female genital tract were higher than blood plasma, and lopinavir and atazanavir achieved low to moderate genital tract concentrations [88]. Efavirenz achieved female genital secretion concentrations <1% blood plasma. In addition, many antiretrovirals are detected in genital secretions within 1–2 hours after the first dose of ART. These results should be used to guide selection of agents for HIV PreP and PEP, and perhaps secondary prevention as well.

Ease of therapy is also an important consideration in choosing an ART regimen for non-occupational postexposure prophylaxis. Two case-controlled studies of non-occupational postexposure prophylaxis following high risk sexual exposures were conducted using tenofovir DF and lamivudine in 44 subjects and the combination of tenofovir DF and emtricitabine in an additional 68 subjects. Subjects in both studies with tenofovir-based dual regimens had higher completion rates of a 28-day postexposure regimen than historical controls taking 2- or 3-drug regimens containing zidovudine (*P *< 0.0001) [89]. Dropout rates during non-occupational postexposure prophylaxis treatment are high [90,91], particularly in cases of sexual assault [92-95]. Although the reasons for discontinuation of therapy may include reassessment of risk exposure and/or intolerable side effects, the evidence of increased adherence with simpler regimens should not be ignored. Finally, it seems clear that health care workers need more education about PEP [96].

## ART and public health reality

There are many mathematical models of the effects of ART on the epidemic, both ART used as PreP [97] or provided to people with established infection [46,98,99]. These models are greatly limited by their assumptions, and none has been subjected to experimental investigation. The biggest questions include adherence, degree of benefit, and population volume served. In other words, are enough people at risk of or infected with the disease receiving the right ART at the right times and for long enough to make a difference? In addition, the public health benefits of ART for people with HIV are up for debate, since ART cannot be readily offered to people with acute HIV infection and people with very advanced disease, since neither group is aware of their status during maximal contagion.

## What if?

We are at a critical juncture for HIV prevention research [2]. It is clear that we cannot simply treat all individuals who become infected. We do not have the tools to make an HIV vaccine [34], and there is no "magic bullet" solution on the horizon. Currently there is intense interest in multi-faceted approaches, but it seems unlikely that behavioral interventions alone will prove sufficient to change the course of the epidemic [3]. The tool currently most readily available is ART, and ART–as PreP, PEP or treatment–will likely play an increasing role in HIV prevention. Indeed, it is possible that the indications for ART treatment will evolve to consider the public health benefit(s) with the same intensity and urgency as the individual therapeutic benefit(s).

Perhaps the most immediate issue facing us is what to do with the things that work. For example, barrier methods (condoms and circumcision) are clearly effective, but they have by no means reached their full prevention potential. The difficulty in rolling out circumcision, especially in countries most greatly affected by HIV, has been a source of great frustration. Similarly, how do we prepare properly for the broad application of ART, should the trials underway demonstrate the anticipated success? And how do we deal with methods that do not work? How do we develop a strategy that recognizes the importance of STDs in HIV transmission without the expectation that treatment of STDs *per se *will alter the course of the epidemic? How do we demonstrate a commitment to vaccine development, short of conducting large-scale clinical trials unlikely to succeed?

These issues can only be properly addressed if the research community works well and creatively with public health leaders and agencies, and this has not always been the case [100]. Given the potential trajectory of HIV prevention, now is the time to address these questions. We have mastered some fundamental tools of HIV prevention, and many more are on the way. In the meantime, we must implement all the tools at our disposal, monitor their successes, and prevent the transmission of HIV.
